# Improving the Selectivity of Metal Oxide Semiconductor Sensors for Mustard Gas Simulant 2-Chloroethyl Ethyl Sulfide by Combining the Laminated Structure and Temperature Dynamic Modulation

**DOI:** 10.3390/s25020525

**Published:** 2025-01-17

**Authors:** Yadong Liu, Siyue Zhao, Lijuan You, Yong Xu, Renjun Si, Shunping Zhang

**Affiliations:** 1 Institute of NBC Defence, Beijing 102205, China; liuyadong202412@163.com (Y.L.); zsy18804169777@126.com (S.Z.); youlijuan8743@163.com (L.Y.); xuzzx0720@sina.com (Y.X.); 2 State Key Laboratory of Material Processing and Die & Mould Technology, Department of Materials Science and Engineering, Huazhong University of Science and Technology, Wuhan 430074, China; rjsi@hust.edu.cn

**Keywords:** MOS sensor, 2-CEES, selectivity, characteristic peak

## Abstract

Insufficient selectivity is a major constraint to the further development of metal oxide semiconductor (MOS) sensors for chemical warfare agents, and this paper proposed an improved scheme combining catalytic layer/gas-sensitive layer laminated structure with temperature dynamic modulation for the Mustard gas (HD) MOS sensor. Mustard gas simulant 2-Chloroethyl ethyl sulfide (2-CEES) was used as the target gas, (Pt + Pd + Rh)@Al_2_O_3_ as the catalytic layer material, (Pt + Rh)@WO_3_ as the gas-sensitive layer material, the (Pt + Pd + Rh)@Al_2_O_3_/(Pt + Rh)@WO_3_ sensor was prepared, and the sensor was tested for 2-CEES and 12 battlefield environment simulation gases under temperature dynamic modulation. The results showed that the sensor only showed obvious characteristic peaks in the resistance response curves to HD under certain conditions (100–400 °C, the highest temperature was held for 1 s and the lowest temperature was held for 2 s), and its peak height reached 6.12, which was far higher than other gases, thus realizing the high selectivity of the MOS sensor to 2-CEES. Meanwhile, the sensor also showed good sensitivity, detection limits, response/recovery times, anti-interference, and stability, which further verified the feasibility of the improved scheme.

## 1. Introduction

HD is a vesicant agent [1] and can be used in artillery shells, rockets, and aerial or ground dispensers. HD is one of the most produced, stockpiled, and used chemical weapons to date, accounting for more than 80% of all known chemical warfare casualties, and is known as the “king of poisons”. HD requires a high level of detection equipment that is fast, sensitive, stable, and accurate.

MOS sensors effectively fulfill the requirements of the detection and early warning for HD, boasting attributes such as swift response time, easy miniaturization, array capability, and portability [2,3,4,5,6,7]. Consequently, MOS sensors have become a promising avenue to develop HD sensors [8,9]. Due to the specificity of HD, experiments were mainly carried out with 2-CEES instead of HD, both of which have similar chemical structures. Yoo [10] synthesized ZnO NPs based on a hydrothermal method, which have good sensing properties for 2-CEES due to their high specific surface area and more active sites. The maximum resistance response to 2-CEES was 15 at a concentration of 1 ppm, and the optimum operating temperature was 250 °C.

However, insufficient selectivity is a major constraint to the further development of MOS sensors [11,12,13]. Researchers have endeavored to enhance selectivity through various means, including temperature modulation technology [14], material morphology and structure control methods [15], metal modification/doping techniques [16], as well as sensor structure improvements [17]. Nevertheless, realizing the high selectivity of MOS sensors for HD is still an unsolved challenge.

In recent years, laminating structures by assembling catalytic layers (such as Co_3_O_4_ [18], SnO_2_ [19], Rh/TiO_2_ [20], and Au [21]) with gas-sensitive materials has been considered a feasible solution to solve the problem and has made progress in the detection of some gases. The main function of the catalytic layer is to catalyze the decomposition of the interfering gas into unreactive or low-reactivity substances before it reaches the gas-sensitive material, thereby improving the selectivity of the sensor for the target gas [22,23]. However, when the composition of the gas mixture to be detected is complex, such as gases in a battlefield environment, the laminated structure still has a limitation in achieving high selectivity.

The sensors can distinguish gases based on the different reaction rates of gases at different temperatures under the temperature dynamic modulation [24], and it can magnify the difference between the signals produced by different gases at different temperatures, thus making it easier to distinguish and identify the signals of the target gas [25]. Temperature dynamic modulation promises to overcome the current drawbacks of laminated MOS sensors.

**Figure 1:**
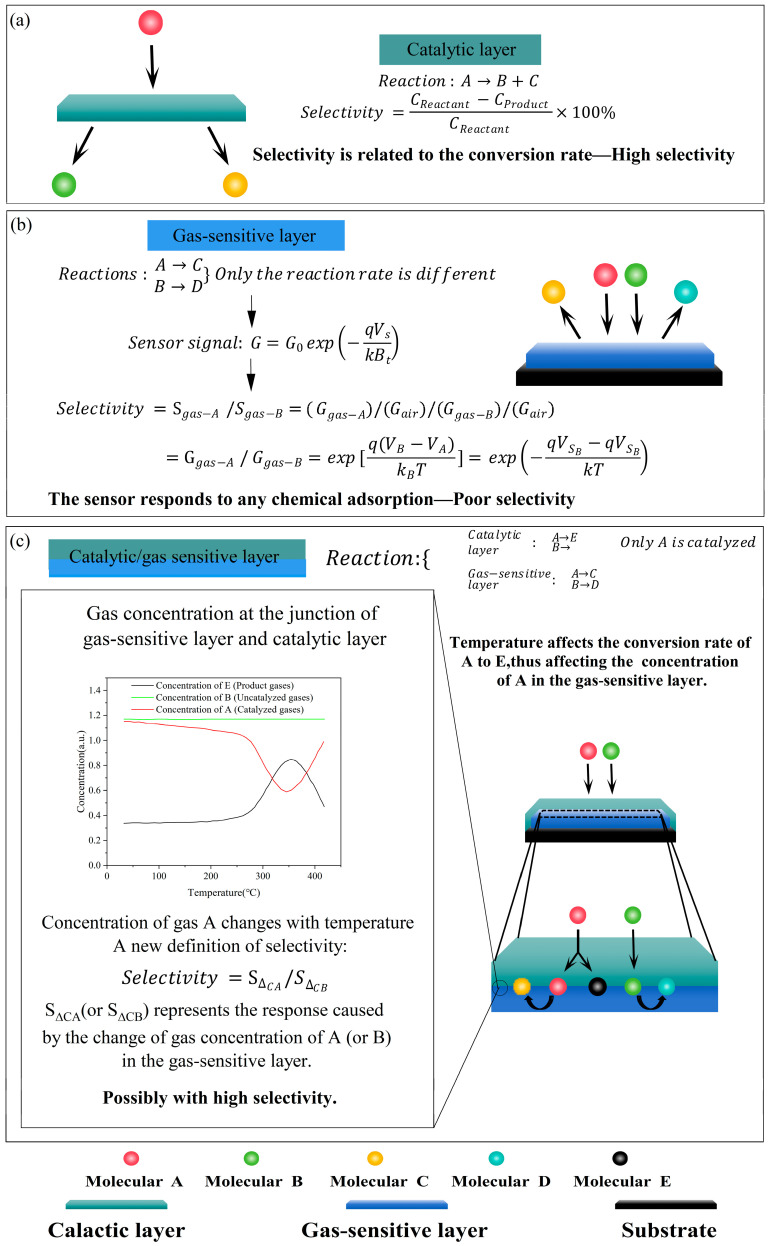
Gas concentration simulation diagram of (**a**) catalytic layer, (**b**) gas-sensitive layer and (**c**) catalytic/gas-sensitive layer.

In this paper, a scheme combining the laminated structure and the temperature dynamic modulation was proposed to improve the selectivity of MOS sensors. The design concept of the scheme is shown in Figure 1. The laminated structure separates the sensitive and conversion elements, using the catalytic layer as the sensitive element and the gas-sensitive layer as the conversion element. As shown in Figure 1a, it is possible to achieve high catalytic selectivity by accelerating key reaction steps to increase the conversion rate. However, the chemisorption of interfering gases leads to charge transfer and resistance changes which generate sensitive signals affecting the selectivity, as shown in Figure 1b. Therefore, we believe that the main reason for the poor selectivity is the lack of isolation between the sensitive element and the conversion element. Temperature dynamic modulation affects the catalytic capacity of the catalytic layer, as well as the concentration of the reacting gas and the product gas within the catalytic layer. For gases that pass directly through the catalytic layer, the concentration of product gas reaching the gas-sensitive layer remains constant with temperature. The gas-sensitive layer detects the reactants and product gases after the catalytic reaction. During such temperature changes, the optimum catalytic capacity is related to the optimum catalytic temperature. Changes in gas concentration may appear as resistance-temperature peaks, thus providing high selectivity, as shown in Figure 1c.

According to the above concept, a (Pt + Rh)@WO_3_/(Pt + Pd + Rh)@Al_2_O_3_ laminated MOS sensor was prepared in this paper using (Pt + Rh)@WO_3_ as the gas-sensitive layer and (Pt + Pd + Rh)@Al_2_O_3_ as the catalytic layer. The high selectivity of this MOS sensor for 2-CEES was realized based on the “characteristic peak” in the conductivity-temperature signals under the temperature dynamic modulation. By calculating the height of the “characteristic peak”, the peak height of the 1 mg/m^3^ 2-CEES was 6.12, while the heights of other 5 mg/m^3^ gases were less than 1. In addition, the sensor also showed good sensitivity, response/recovery times, anti-interference, and stability to 2-CEES.

## 2. Materials and Methods

### 2.1. Preparation of Layer Materials

#### 2.1.1. Preparation of (Pt + Rh)@WO_3_ Slurry

We dispersed 10 mmol WCl_6_ in 50 mL n-heptane and stirred it for 1 h to obtain the precursor, which was reacted at 160 °C for 24 h. After drying, it was washed twice with a mixture of anhydrous ethanol and dichloromethane and twice with pure water, then put into an oven to be burned at 450 °C for 2 h to obtain the WO_3_ matrix powder. We dispersed 2 g WO_3_ powder in 20 mL anhydrous ethanol, added calculated 0.01 g/mL H_2_PtCl_6_-6H_2_O ionic solution and 0.01 g/mL RhCl_3_-3H_2_O ionic solution, stirring for 1 h. Then, we placed it in the oven to dry at 60 °C and obtained 0.5 wt% Pt^4+^, Rh^3+^-modified WO_3_ powder. The modified WO_3_ powder was obtained by the above steps. A total of 3 g (Pt + Rh)@WO_3_ powder was added to 9 mL nano-alumina dispersion and ball-milled at 400 rpm for 4 h to obtain (Pt + Rh)@WO_3_ slurry.

#### 2.1.2. Preparation of (Pt + Pd + Rh)@Al_2_O_3_ Slurry

A total of 1 g of γ-Al_2_O_3_ and calculated 0.01 g/mL H_2_PtCl_6_-6H_2_O ionic solution, 0.01 g/mL PdCl_2_ ionic solution, and 0.01 g/mL RhCl_3_-3H_2_O ionic solution were put into the ball-milling jar to obtain 0.5 wt% Pt^4+^, Pd^2+^, and Rh^3+^-modified γ-Al_2_O_3_. Then, 10 mL of anhydrous ethanol was added to the ball-milling jar. The ball milling was performed at 400 rpm for 4 h. The (Pt + Pd + Rh)@Al_2_O_3_ slurry was obtained by the above steps.

### 2.2. Preparation of the Catalytic Layer/Gas-Sensitive Layer MOS Sensor

**Figure 2:**
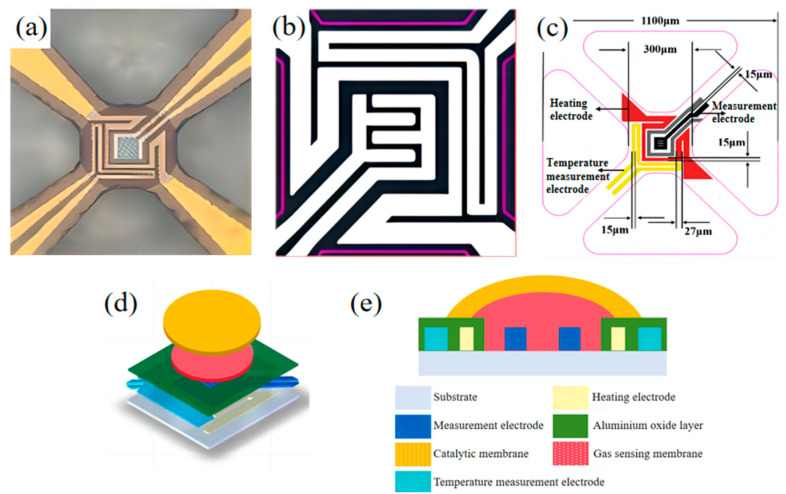
(**a**–**c**) schematic diagrams, and (**d**,**e**) physical diagram of the sensor chip.

Figure 2a–c illustrate the MOS sensor chip. Figure 2a presents the chip’s physical diagram, and Figure 2b details the electrode design. The electrodes were categorized into heating, temperature measurement, and measurement electrodes, with their positions and size parameters shown in Figure 2c. Using an electrofluidic microspray process, a 60 µm needle first sprayed (Pt + Rh)@WO_3_ slurry onto the electrode site on the zirconia microthermal plate chip. To prevent interpenetration between the two layers, the gas-sensitive layer was dried at 100 °C for 1 h. Next, the (Pt + Pd + Rh)@Al_2_O_3_ slurry was applied using a 100 µm needle, as depicted in Figure 2d,e. Finally, the chip would be placed in a muffle furnace at 350 °C and 550 °C for 2 h each to produce the (Pt + Rh)@WO_3_/(Pt + Pd + Rh)@Al_2_O_3_ laminated sensor.

First, an electrofluidic microspray process was utilized to spray Pt@SnO_2_ slurry at the measurement electrode site of the gas-sensitive layer chip and CeMnOx slurry at the measurement electrode site of the catalytic layer chip using a 100 μm aperture needle. Then, the sprayed chips were fired in a muffle furnace at 350 °C and 550 °C for 2 h each. Finally, the fired chips were soldered and packaged to make MOS sensors that could be used for testing.

### 2.3. Test Platform

**Figure 3:**
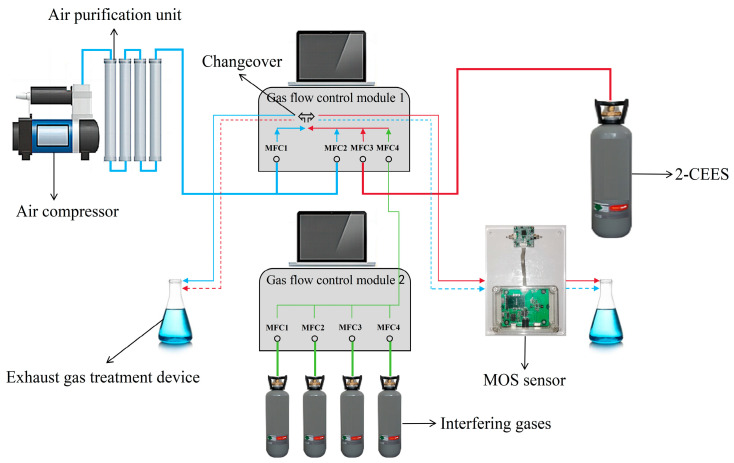
Test platform.

The test platform is shown in Figure 3. Air is supplied by an air compressor, and through the air purification device, the purified air is divided into two paths, MFC1 and MFC2 (referred to as 1-MFC1 and 1-MFC2), in the gas flow control module 1. 2-CEES is supplied by gas cylinders, and enters MFC3 (referred to as 1-MFC3) in gas flow control module 1. The interfering gases are supplied by gas cylinders, and the interfering gases enter MFC1, MFC2, MFC3, and MFC4 (referred to as 2-MFC1, 2-MFC2, 2-MFC3, and 2-MFC4) in gas flow control module 2. Each of the above gases can be adjusted for flow size. The interfering gases are mixed after passing through 2-MFC1, 2-MFC2, 2-MFC3, and 2-MFC4, and the mixed interfering gases enter MFC4 (referred to as 1-MFC4) in the gas flow control module 1. After mixing, 1-MFC2+1-MFC3+1-MFC4 will be formed into the 2-CEES path, and 1-MFC1 will be the air path. Switching of the 2-CEES path and air path can be realized by controlling the changeover, which alternately enters into the MOS sensor and the exhaust gas treatment device.

The total flow of the test gas is 1000 sccm. Considering that water and other gases in the environment have a large effect on MOS sensors, it is not conducive to accurate testing of performance. Therefore, sufficient desiccant and absorbent are added to the air purification unit in the test platform. The exposure time of the sensor in the gas varies according to the needs of the test.

2-CEES was purchased from the Clean Energy Technology Co., Ltd. (Shanghai, China) at a concentration of 98 ppm with nitrogen as the carrier gas. Interfering gases were purchased from the Clean Energy Technology Co., Ltd. at a concentration of 80 ppm and the carrier gas was nitrogen.

## 3. Results and Discussion

### 3.1. Characterization of Material

**Figure 4:**
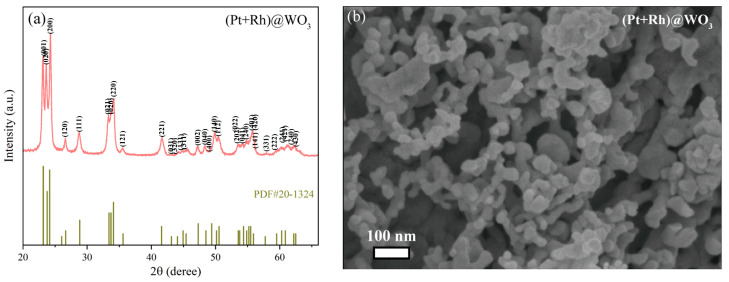
(**a**) XRD pattern and (**b**) SEM image of (Pt + Rh)@WO_3_ powder.

Figure 4a shows the XRD pattern of (Pt + Rh)@WO_3_ powders, which was obtained using a Philips X’ Pert diffractometer (2θ from 10° to 80°, λ = 1.5406 Å) with Cu Kα radiation. It can be seen that the (Pt + Rh)@WO_3_ was of good purity and crystallinity and had a few peaks of extra impurities. WO_3_ is indexable to the appropriate standard card (20-1324). Due to the small amount of Pt^4+^ and Rh^3+^ modification, the figure does not clearly reveal information related to these modification elements. Figure 4b presents the SEM image of (Pt + Rh)@WO_3_ powder. The structure of (Pt + Rh)@WO_3_ appears as an accumulation of uniform nanoparticles. The synthesized (Pt + Rh)@WO_3_ nanoparticles have a diameter of approximately 20 nm.

### 3.2. Definition of Characteristic Peak

**Figure 5:**
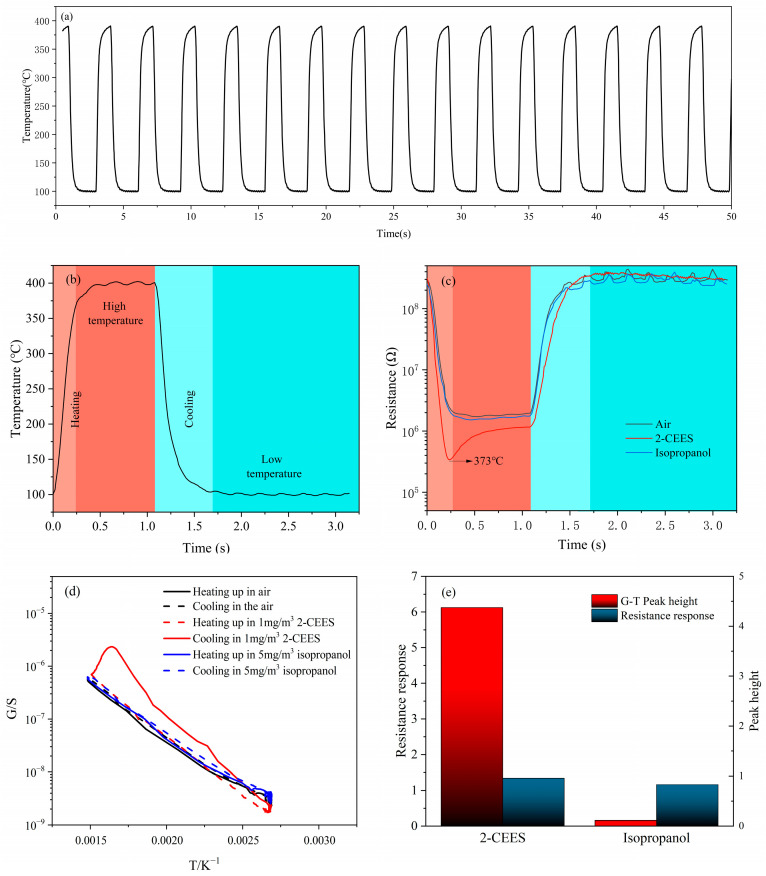
(**a**) Temperature modulation ability of the sensor, (**b**) the T-t curve, (**c**) the R-t curves, (**d**) the G−T curves for air, 1 mg/m^3^ 2-CEES and 5 mg/m^3^ isopropanol, and (**e**) the G−T peak heights and resistance responses to 1 mg/m^3^ 2-CEES and 5 mg/m^3^ isopropanol.

Figure 5a demonstrates the temperature variation of the sensor under the parameters (100 °C–400 °C, high temperature was held for 1 s, low temperature was held for 2 s). The noise of the sensor at the maximum temperature of 400 °C is ±2 °C, and a single cycle of temperature change has a period duration of less than 3.2 s, which demonstrates the sensor’s ability to regulate temperature very consistently and quickly. A single cycle of the temperature–time (T-t) curves is shown in Figure 5b. Figure 5c demonstrates the resistance–time (R-t) curves of the sensor to air, 1 mg/m^3^ 2-CEES, and 5 mg/m^3^ isopropanol in a single cycle. The resistance of the sensor decreased when the sensor was exposed to 2-CEES and isopropanol compared to air. In addition, a “sharp peak” in the resistance response of 2-CEES can be observed during the heating section that is significantly different from air and isopropanol, which we call the “characteristic peak”.

The presence of the characteristic peak provides a possible reference for achieving high selectivity of the sensor for 2-CEES. In order to quantify the characteristic peak, a rational calculation method is required. By processing various aspects of the measured data, it was concluded that the magnitude of the characteristic peak could be reasonably quantified by combining the T-t curves and the R-t curves into G−T curves (G is commonly used as the symbol for conductance, which stands for the reciprocal of resistance). Figure 5d shows the G−T curves of air, 1 mg/m^3^ 2-CEES, and 5 mg/m^3^ isopropanol. The G−T curve of 5 mg/m^3^ isopropanol is similar to that of air, whereas the curve of 1 mg/m^3^ 2-CEES gas displays a clear peak. The height of this peak can be used to quantify the characteristic peak.

If the optimal catalytic temperature of the catalytic layer for 2-CEES corresponded to Tc, which was the highest point of the peak of the G−T curve, the ratio of the conductance at the highest point of the peak of the G−T curve at this point in the 2-CEES to the bottom of the peak at the corresponding temperature could be calculated as follows:

(1) $$S_{\mathrm{Gas}}=\frac{R_{\left(\mathrm{Gas},C,\mathrm{Tc}\right)}^{-1}}{R_{\left(\mathrm{Gas},H,\mathrm{Tc}\right)}^{-1}}=\frac{R_{\left(\mathrm{Gas},H,\mathrm{Tc}\right)}}{R_{\left(\mathrm{Gas},C,\mathrm{Tc}\right)}}$$

where R_(Gas, C, Tc)_ and R^−1^_(Gas, C, Tc)_ denote the resistance and the reciprocal of this resistance during the cooling section to Tc in 2-CEES, respectively; R_(Gas, H, Tc)_, R^−1^_(Gas, H, Tc)_ denoted the resistance and the reciprocal of the resistance of the sensor during the heating section to Tc; and S_Gas_ denoted the intrinsic peak height of 2-CEES. Similarly, the intrinsic peak height of air at Tc was:

(2) $$S_{\mathrm{Air}}=\frac{R_{\left(\mathrm{Air},C,\mathrm{Tc}\right)}^{-1}}{R_{\left(\mathrm{Air},H,\mathrm{Tc}\right)}^{-1}}=\frac{R_{\left(\mathrm{Air},H,\mathrm{Tc}\right)}}{R_{\left(\mathrm{Air},C,\mathrm{Tc}\right)}}$$

The meaning of each parameter was similar to that in a 2-CEES atmosphere, R_(Air, C, Tc)_, R^−1^ _(Air, C, Tc)_ denoted the resistance and the reciprocal of this resistance when cooling to Tc in air, and R_(Air, H, Tc)_, R^−1^ _(Air, H, Tc)_ denoted the resistance of the sensor and the reciprocal of the corresponding resistance when heating to Tc, respectively. Therefore, the G−T peak height of 2-CEES was:

(3) $$S_{\mathrm{Peak}}=S_{\mathrm{Gas}}-S_{\mathrm{Air}}$$

The G−T peak heights and resistance responses of 1 mg/m^3^ 2-CEES and 5 mg/m^3^ isopropanol were calculated as illustrated in Figure 5e. It can be observed from the figure that the peak heights for 1 mg/m^3^ 2-CEES and 5 mg/m^3^ isopropanol are measured at 6.12 and 0.16, respectively, while their corresponding resistance responses were determined as 1.34 and 1.16.

### 3.3. Selectivity

**Figure 6:**
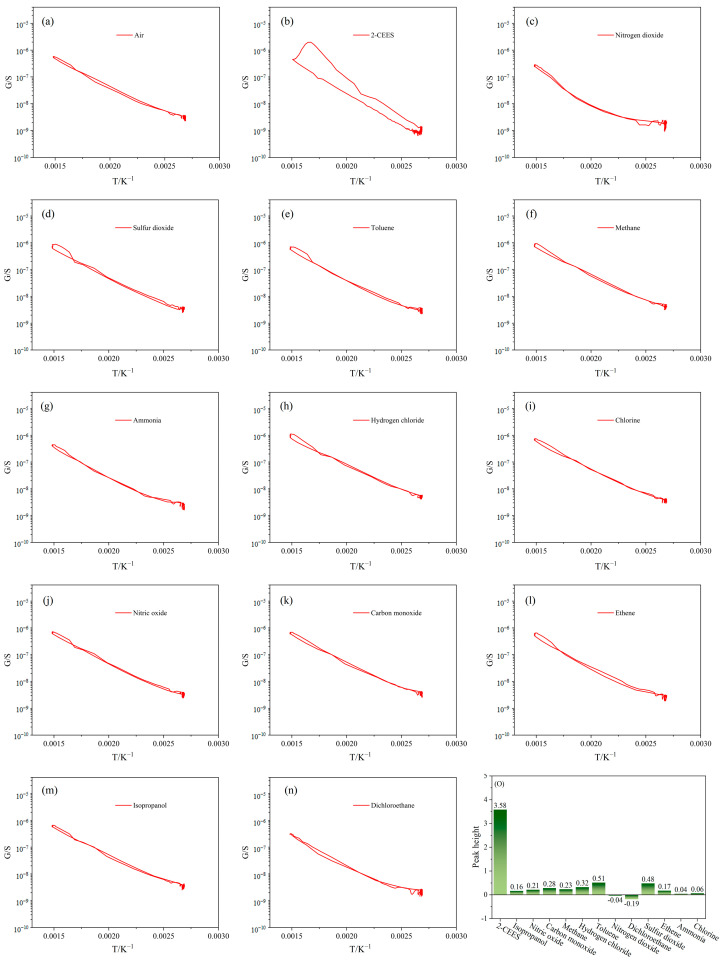
(**a**–**n**) G−T curves of the (Pt + Rh)@WO_3_/(Pt + Pd + Rh)@Al_2_O_3_ sensor to different gases, and (**o**) their G−T characteristic peak heights.

Figure 6a–n presents the G−T curves to 1 mg/m^3^ 2-CEES and various other 5 mg/m^3^ gases. It is clear that the sensor shows no distinct characteristic peak signals for any gases except 2-CEES on the G−T curves. This is possibly a result of the distinctive catalytic ability of the (Pt + Pd + Rh)@Al_2_O_3_ catalytic layer for 2-CEES. As shown in Figure 6o, 2-CEES reached a peak height of 6.12. However, the peak heights of the other gases were less than 1. This indicates that the scheme is effective in achieving high selectivity of the sensor for 2-CEES.

Since the peak height was used in this paper to evaluate the selectivity of the sensor for 2-CEES, we adopted the ratio of the peak height of 2-CEES (S_2-CEES Peak_) and the peak height of the interfering gas (S_Intering gas-Peak_) as the selectivity factor (S_i_) when 2-CEES was mixed with other interfering gases. The peak height of 2-CEES at 1 mg/m^3^ was 6.12 and the highest peak height of the 12 interfering gases at 5 mg/m^3^ was toluene (0.51). The selectivity factor of the sensor for 2-CEES at this concentration exceeded 12.

(4) $$S_{i}=\frac{S_{2\text{-}\mathrm{CEES}\text{-}\mathrm{Peak}}}{S_{\mathrm{Intering}\;\mathrm{gas}\text{-}\mathrm{Peak}}}$$

### 3.4. Sensitivity and Detection Limit

To standardize the calculations, the resistance response of the sensor (SG) is defined as follows: “H” represents the heating processes, respectively. R_(Air, H, 373 °C)_ refers to the sensor’s resistance value when cooled to 150 °C in air. Similarly, R_(Gas, H, 373 °C)_ refers to the resistance value when the sensor cooled to 150 °C in the test gas.

(5) $$S_{G}=\frac{R_{\left(\mathrm{Air},H,373{}^{\circ}C\right)}-R_{\left(\mathrm{Gas},H,373{}^{\circ}C\right)}}{R_{\left(\mathrm{Gas},H,373{}^{\circ}C\right)}}$$

**Figure 7:**
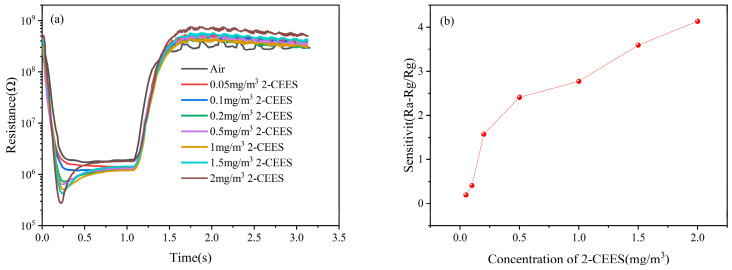
(**a**) resistance response, and (**b**) sensitivity of 2-CEES at different concentrations.

Figure 7a,b show the resistance response and sensitivity of 2-CEES at different concentrations, respectively. It can be seen that the resistance correspondingly increased with the increase in concentration and the sensitivity reached more than 1.57 when the concentration reached more than 0.2 mg/m^3^, and the sensitivity was 4.13 at a concentration of 2 mg/m^3^, indicating that the sensor had high sensitivity to 2-CEES. It is worth noting that when the concentration was as low as 0.05 mg/m^3^, the resistance of 2-CEES was almost the same as that of air, so the detection limit of the sensor for 2-CEES was about 0.05 mg/m^3^.

### 3.5. Response/Recovery Times

**Figure 8:**
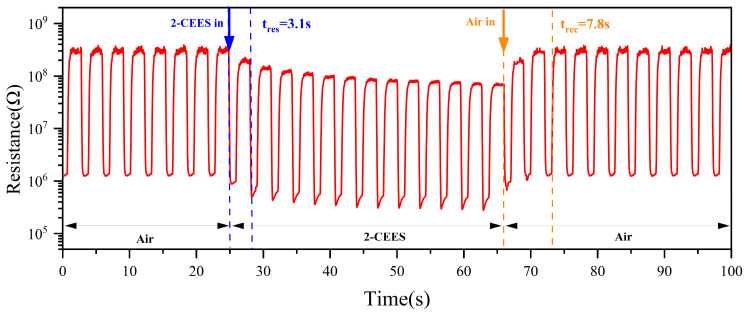
Response/recovery times of the sensor to 1 mg/m^3^ 2-CEES.

The test of the sensor to 1 mg/m^3^ 2-CEES is shown in Figure 8. After the entry of 2-CEES, a more obvious characteristic peak was observed after 3.1 s, so the response time was about 3.1 s. After the entry of air, the resistance returned to a steady state after 7.8 s, so the recovery time was about 7.8 s.

### 3.6. Anti-Interference

Based on the results of a study published in 1999 by the Chemistry Department of the Naval Research Laboratory (NRL) on the composition of gases in the battlefield environment [26], a total of 12 interfering gases have been in the simulated battlefield environment in the experiment. They were carbon monoxide, nitric oxide, nitrogen dioxide, methane, ethene, sulfur dioxide, toluene, dichloroethane, ammonia, hydrogen chloride, chlorine, and isopropanol.

The anti-interference capability of the sensor was examined. A total of 12 interfering gases were selected to assess the sensor’s anti-interference abilities against 2-CEES. The experiments were conducted in four groups, with 1 mg/m^3^ 2-CEES and 5 mg/m^3^ interfering gases added to each group, as shown in Table 1.

**Figure 9:**
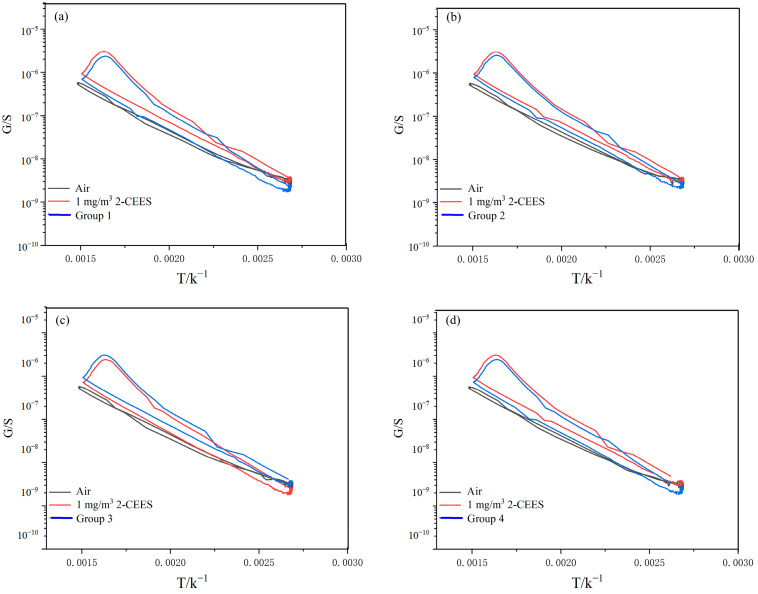
(**a**–**d**) G−T curves of the (Pt + Rh)@WO_3_/(Pt + Pd + Rh)@Al_2_O_3_ sensor for air, 1 mg/m^3^ 2-CEES, and four groups of gases.

Figure 9a–d shows the G−T curves of the sensor for 2-CEES and four groups of gases, and the shape of the G−T curve for 2-CEES did not change significantly with the addition of the interfering gas. This indicates that the interfering gas did not significantly deplete the 2-CEES during the mixing stage of the interfering gas and 2-CEES. By calculating the peak heights of the four groups of gases, the peak heights of 2-CEES under the interference of the above gases varied in the range of 5.89–6.15, which was still significantly higher than that of the interfering gases. It shows that the sensor has good anti-interference ability for 2-CEES.

### 3.7. Stability

**Figure 10:**
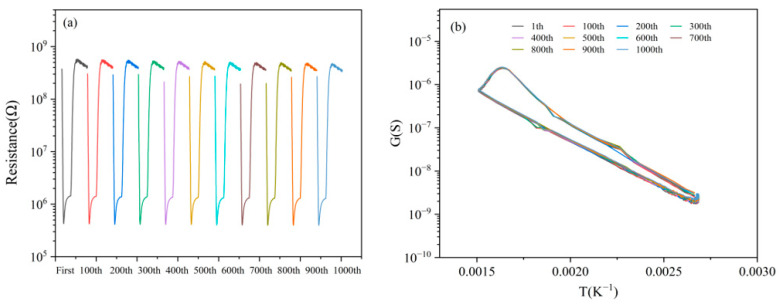
Stability of (**a**) resistances and (**b**) peak heights for 1 mg/m^3^ 2-CEES.

We conducted the following experiments to verify the sensor’s stability. The sensor was tested 1000 times continuously for 1mg/m^3^ 2-CEES, with data recorded every 100 cycles. The results are shown in Figure 10a,b. The resistance of the 1000th cycle is not significantly different from that of the first cycle, and the characteristic peak of 2-CEES is almost unchanged, with the peak heights fluctuating between 6.07 and 6.22.

Table 2 summarizes some of the research advances on MOS sensors for 2-CEES. By comparing the experimental results of this paper with those of other scholars, three main aspects are summarized. First, we performed tests for anti-interference, which are rarely reported in the current literature. The sensor has relatively good selectivity, sensitivity, detection limits, and response/recovery times for HD compared to other sensors reported in the literature, especially with regard to the selectivity test, and we compared 12 gases, which is more than most of the 2-CEES sensors currently available are able to test. Finally, with regard to sensor stability, we only tested 1000 consecutive cycles and did not perform stability tests over a large time span (e.g., over months), which is a task to be addressed in the future.

### 3.8. Highly Selective Sensing Mechanisms

Based on the oxygen adsorption model, the sensing mechanism of (Pt + Rh)@WO_3_ gas-sensitive layer for 2-CEES was discussed. It is well known that the layer surface usually is covered with chemisorbed oxygen ions, including $O_{2}^{-}$, O^−^, and O^2−^. As the temperature increases, the reaction can be described as follows:

(6) $$O_{2}\left(\mathrm{ads}\right)+e^{-}↔O_{2}^{-}\left(\mathrm{ads}\right)$$

(7) $$O_{2}^{-}\left(\mathrm{ads}\right)+e^{-}↔{2O}^{-}\left(\mathrm{ads}\right)$$

(8) $$O^{-}\left(\mathrm{ads}\right)+e^{-}↔O^{2-}\left(\mathrm{ads}\right)$$

$O_{2}^{-}$, O^−^, and O^2−^ are separately formed in 25~150 °C, 150~300 °C and more than 300 °C. The interaction between 2-CEES and (Pt + Rh)@WO_3_ layer is as follows:

(9) $$O_{2}^{-}+2S\left({\mathrm{CH}}_{2}{\mathrm{CH}}_{3}\right)\left({\mathrm{CH}}_{2}{\mathrm{CH}}_{2}\mathrm{Cl}\right)=2S\left({\mathrm{CH}}_{2}{\mathrm{CH}}_{3}\right)\left({\mathrm{CH}}_{2}{\mathrm{CH}}_{2}\mathrm{Cl}\right)O+e^{-}$$

(10) $$O^{-}+S\left({\mathrm{CH}}_{2}{\mathrm{CH}}_{3}\right)\left({\mathrm{CH}}_{2}{\mathrm{CH}}_{2}\mathrm{Cl}\right)=S\left({\mathrm{CH}}_{2}{\mathrm{CH}}_{3}\right)\left({\mathrm{CH}}_{2}{\mathrm{CH}}_{2}\mathrm{Cl}\right)O+e^{-}$$

(11) $$O^{2-}+S\left({\mathrm{CH}}_{2}{\mathrm{CH}}_{3}\right)\left({\mathrm{CH}}_{2}{\mathrm{CH}}_{2}\mathrm{Cl}\right)=S\left({\mathrm{CH}}_{2}{\mathrm{CH}}_{3}\right)\left({\mathrm{CH}}_{2}{\mathrm{CH}}_{2}\mathrm{Cl}\right)O+{2e}^{-}$$

2-CEES would release electrons on the surface of (Pt + Rh)@WO_3_, leading to a decrease in the resistance of (Pt + Rh)@WO_3_. In this study, Pt and Rh were chosen for modification due to the following reasons: (1) It has been shown that 2-CEES molecules tended to adsorb on the WO_3_ surface via Cl-O bonds. In contrast, 2-CEES molecules preferred to adsorb on WO_3_ NSs/Pd hybrids via Cl-Pd bonding and exhibited higher negative adsorption energies, and the same were higher than the adsorption energies for hydrogen sulfide, ammonia, and nitrogen dioxide [27]. Therefore, we speculate that (Pt + Rh)@WO_3_ had a good adsorption capacity for 2-CEES molecules when it was mixed with other interfering gases. (2) Under normal conditions, when the oxide catalyst surface is alkaline, 2-CEES undergoes oxidation reactions to produce S(CH_2_CH_3_)(CH_2_Cl_2_)O. By incorporating Pt and Rh, an alkaline surface is formed on WO_3_. This alkaline surface can enhance the catalytic activity for the oxidation of 2-CEES to S(CH_2_CH_3_)(CH_2_CH_2_Cl)O, thus improving sensitivity towards 2-CEES.

**Figure 11:**
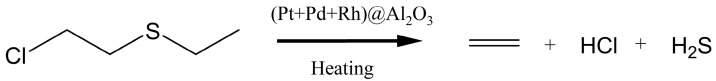
Hydrodesulfurization and hydrodechlorination reactions of 2-CEES molecules catalyzed by (Pt + Pd + Rh)@Al_2_O_3_.

Under heating conditions, 2-CEES will produce hydrodesulfurization and hydrodechlorination reactions as they pass through the (Pt + Pd + Rh)@Al_2_O_3_ catalytic layer, generating ethylene, hydrogen chloride, and hydrogen sulfide, as shown in Figure 11 [31]. 2-CEES contains a thioether group in which sulfur is in a lower oxidation state and can be oxidized to a higher oxidation state, exhibiting higher reducing properties than ethylene, hydrogen chloride, and hydrogen sulfide. So the response between 2-CEES and the gas-sensitive layer was much larger than that between its reaction products and the gas-sensitive layer.

**Figure 12:**
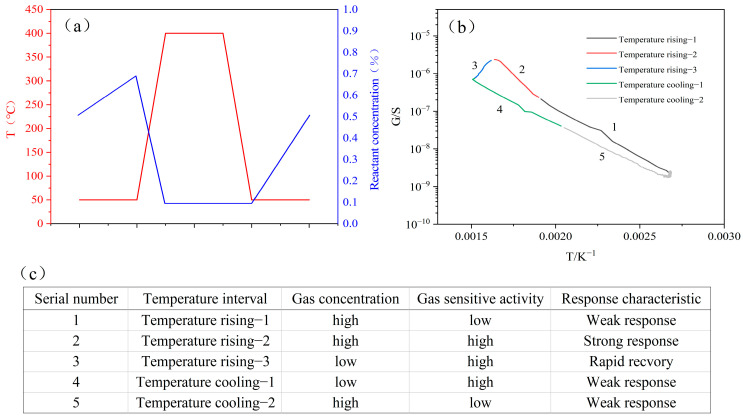
(**a**) the concentration changes of reactant gas on the surface of the gas-sensitive layer, (**b**) the G−T curve of 2-CEES, and (**c**) the response characteristics of each stage.

Finally, the generation of the characteristic peak was considered. The single cycle is divided into five sections: heating intervals 1 to 3, and cooling intervals 1 and 2, as illustrated in Figure 12b. Each section is numbered from 1 to 5.

In interval 1, as the temperature rises, the catalytic activity of the layer increases. Gas reactants begin decomposing as they pass through the catalytic layer, the gas concentration on the gas-sensitive layer surface increases, and the sensor produces a resistance response.

In interval 2, when the temperature reaches the optimal catalytic point (373 °C), the resistance response of the gas-sensitive layer reaches its maximum.

In interval 3, as the temperature rises further, the catalytic ability of the catalytic layer weakens. The amount of decomposed gas reactants decreases, the gas concentration on the gas-sensitive layer surface decreases, and resistance starts to recover, i.e., the appearance of characteristic peaks.

In interval 4, when the temperature hits its peak, it begins to drop. The catalytic layer gradually loses its catalytic activity, the gas-sensitive layer detects a decrease in gas reactant concentration, and the resistance response weakens.

In interval 5, when the temperature continues to decrease, the catalytic layer does not have catalytic activity. At this time, the gas reactants can diffuse through the layer, the concentration of the gas reactants is detected by the gas-sensitive layer, and the resistance response is weak. In a single cycle, the simulation results and states of the reactant concentrations of each stage are shown in Figure 12a,c.

## 4. Conclusions

In the experiments of this paper, the catalytic efficiency of the (Pt + Pd + Rh)@Al_2_O_3_ catalytic layer for 2-CEES was different at different temperatures, and an optimal catalytic temperature existed. The dynamic modulation of temperature leads to changes in the concentration of reaction products after 2-CEES passes through the catalytic layer when it reaches the surface of (Pt + Rh)@WO_3_ gas-sensitive layer, and the gas-sensitive layer could detect 2-CEES and reaction products after the catalytic reaction. The gas-sensitive layer could convert the concentration change of 2-CEES, which firstly increased and then decreased in the process of heating, into the peak R-T signal, i.e., the specific response, so as to realize the high selectivity to 2-CEES. The specific response, as a feature reflected in the response curve of the MOS sensor to the target gas, has a strong resistance to interference and is suitable for gases such as HD that require accurate identification.

## Figures and Tables

**Table 1:** Gas grouping situation.

Group Number	Gas Composition			
	1 mg/m^3^	5 mg/m^3^		
1	2-CEES	carbon monoxide	nitric oxide	nitrogen dioxide
2	2-CEES	methane	ethene	toluene
3	2-CEES	hydrogen chloride	chlorine	ammonia
4	2-CEES	sulfur dioxide	dichloroethane	Isopropyl alcohol

**Table 2:** MOS sensors used for detection of 2-CEES.

Basic Metal Oxide Semiconductor	Test Concentration	Tem. (°C)	Sel. ()	Sen. (Ra-Rg/Rg)	DL.	R/r T. (s)	AI.	Sta. (days)	Ref.
(Pt + Rh)@WO_3_/ (Pt + Pd + Rh)@Al_2_O_3_	182 ppb	373	>12	15.3	9 ppb	3.1/7.8	12 gases	-	
WO_3_ NSs/Pd	700 ppb	260	>8	7.5	15 ppb	9/92	-	30	[27]
Al-doped ZnO NPs	20 ppm	500	>15	954.2	-	-/ >250	-	-	[28]
ZnO NPs	1 ppm	250	>4.4	15	0.4 ppm	-	-	-	[10]
Ru loaded CdSnO_3_	4 ppm	350	>8.59	62.12	-	5/185	-	-	[29]
Crosslinked WO_3_ nanonet	50 ppm	217	>11	57	300 ppb	1/-	-	90	[30]

Abbreviations in the table: temperature (Tem.), selectivity (Sel.), sensitivity (Sen.), detection limit (DL.), response/recovery time (R/r T.), anti-interference (AI.), stability (Sta.), reference (Ref.).

## Data Availability

Data are contained within the article.
